# Evidence for the use of complementary and alternative medicines during fertility treatment: a scoping review

**DOI:** 10.1186/s12906-018-2224-7

**Published:** 2018-05-15

**Authors:** Skye A. Miner, Stephanie Robins, Yu Jia Zhu, Kathelijne Keeren, Vivian Gu, Suzanne C. Read, Phyllis Zelkowitz

**Affiliations:** 1 Department of Sociology, McGill University, Room 712, Leacock Building, 855 Sherbrooke Street West, Montreal, QC H3A 2T7 Canada; 2 Department of Psychiatry, Jewish General Hospital, 4333 Chemin de la Cote-Ste-Catherine, Room 223, Montreal, QC H3T 1E4 Canada; 3 Department of Dentistry, McGill University, 2001 McGill College Ave, Montreal, QC H3A 1G1 Canada; 4 Department of Medicine, University of British Columbia Medical School, 2275 Laurel Street, 10th Floor, British Columbia, BC V5Z 1M9 Canada; 5 Lady Davis Institute, 3755 Chemin de la Cote-Ste-Catherine, Montreal, QC H3T 1E2 Canada; 6 Department of Psychiatry, McGill University, Ludmer Research and Training Building, 1033 Pine Ave. West., Montreal, QC H3A 1A1 Canada

**Keywords:** Complementary and alternative medicine, Infertility treatment, Mental health, Acupuncture, Reproductive health, Scoping review

## Abstract

**Background:**

Complementary and alternative medicines (CAM) are sometimes used by individuals who desire to improve the outcomes of their fertility treatment and/or mental health during fertility treatment. However, there is little comprehensive information available that analyzes various CAM methods across treatment outcomes and includes information that is published in languages other than English.

**Method:**

This scoping review examines the evidence for 12 different CAM methods used to improve female and male fertility outcomes as well as their association with improving mental health outcomes during fertility treatment. Using predefined key words, online medical databases were searched for articles (*n* = 270). After exclusion criteria were applied, 148 articles were analyzed in terms of their level of evidence and the potential for methodological and author bias.

**Results:**

Surveying the literature on a range of techniques, this scoping review finds a lack of high quality evidence that complementary and alternative medicine (CAM) improves fertility or mental health outcomes for men or women. Acupuncture has the highest level of evidence for its use in improving male and female fertility outcomes although this evidence is inconclusive.

**Conclusion:**

Overall, the quality of the evidence across CAM methods was poor not only because of the use of research designs that do not yield conclusive results, but also because results were contradictory. There is a need for more research using strong methods such as randomized controlled trials to determine the effectiveness of CAM in relation to fertility treatment, and to help physicians and patients make evidence-based decisions about CAM use during fertility treatment.

**Electronic supplementary material:**

The online version of this article (10.1186/s12906-018-2224-7) contains supplementary material, which is available to authorized users.

## Background

People facing infertility concerns are increasingly turning to the use of assisted reproductive technologies. However, successful treatment outcomes are far from certain because 68.5% of IVF cycles do not result in a live birth [1]. These high failure rates lead many couples to look for ways to improve their chances of achieving conception. Complementary and alternative medicines (CAM) purportedly offer couples a way to improve outcomes and/or decrease stress and anxiety levels during treatment [2–4]. CAM are also used to incorporate cultural traditions of health and fertility as well as increase feelings of hope and control during a biomedicalized fertility treatment plan [5]. Some patients also use CAM as an alternative to assisted reproductive technologies, although most fertility patients use CAM in addition to biomedical fertility treatment [6, 7]. However, little comprehensive information exists on the effectiveness of these methods, leaving both patients and physicians lacking the necessary knowledge to make evidence-based decisions about whether to use CAM during fertility treatment.

CAM is defined by the Centers for Disease Control (CDC) as a “group of diverse medical and health care systems, practices, and products that are not presently considered to be part of conventional medicine” [8]. The current definition of CAM is broad but inclusive, encompassing acupuncture, body work (e.g. massage), energy healing (e.g. reiki), herbal medicines (e.g. naturopathy), mind-body techniques (e.g. meditation, yoga), and traditional medicines (e.g. Chinese medicine). Some CAM relies on alternative practitioners to administer these methods (e.g. acupuncture) while others require a change in behavior by the individual using CAM (e.g. meditation). The various types of CAM offered, along with the increasing market for alternative fertility products, makes it pertinent to have a broader understanding of the effectiveness of these treatments.

The stigma, costs, and uncertainty associated with biomedical fertility treatments often entice those who are having problems conceiving to use CAM as a first line of treatment before engaging in more medically invasive treatments [6, 9]. Recent studies suggest that fertility patients often see CAM methods as a safe and effective way to increase their fertility [2], and are thus willing to try alternative treatments and remedies to supplement conventional approaches to fertility treatment. The holistic approach that many CAM methods purport also offers a way for current fertility patients to offset some potential negative side-effects of biomedical treatments [2]. Additionally, the patient-centered focus of CAM provides fertility patients a feeling of control over the treatment process [4, 7]. While these “fertility-enhancing” treatments are often sold and marketed to couples attempting to conceive naturally or through biomedical processes, their effectiveness is often unknown.

CAM is not only used to increase fertility, but also to decrease patient levels of stress and anxiety during the taxing process of treatment [2, 10]. A lack of knowledge surrounding CAM’s effectiveness and potential negative effects along with the belief that CAM makes one psychologically stronger contributes to the prevalence of CAM use during fertility treatment; 29–91% of fertility patients report using a CAM method during treatment [4].

The wide range of fertility patients reporting CAM use is partially due to differing definitions of CAM in the literature. The review of CAM by Rayner, Willis and Burgess [4] reports on eight different studies and found the highest prevalence of use was 91%. A 2013 study by Clark reports a similar prevalence of CAM use by fertility patients in the US where 91.3% reported using CAM with 73% of these patients believing CAM had beneficial effects on their fertility [10]. The high rates of CAM endorsement may be due to a number of factors: their broad definition (e.g. including exercise as a CAM method), and the sample bias (i.e. those who use CAM are potentially more likely to answer a survey examining CAM-seeking behaviors). On the other hand, more restrictive definitions of CAM that exclude more common practices such as prayer, or consider only a subset of CAM treatments such as herbal or alternative medicine, report a lower prevalence of use 8.3–29% [11, 12], suggesting that reported prevalence of CAM use is highly dependent on definition.

Rayner, Willis and Burgess [4] found that the most commonly used CAM methods for promoting fertility include herbal medicine, acupuncture and nutritional advice/supplements while the most rarely used include religious intervention, spiritual healing^1^, fertility accessories (e.g. necklaces, rocks) and changes in attire/sexual practices. The differences in popularity of these methods may be due not only to patients’ personal preferences, but also to the perception that some methods have better outcomes. However, the benefits of CAM have not been systematically evaluated across CAM methods as most comprehensive reviews focus on why people use CAM, and prevalence and types of CAM used.

This paper provides a scoping review of the potential benefits of various CAM methods, while describing the quality of that evidence. This paper advances the literature in this field by a) looking at a wider variety of CAM methods by including methods such as Ayurveda, herbal medicine, osteopathy, and hypnosis that have not been previously summarized in the literature; b) assessing CAM effectiveness in relation to female and male factor infertility as well as mental health outcomes; c) reviewing articles in multiple languages including Chinese, English, French and Korean; d) not limiting the evidence to randomized clinical trials or previously compiled meta-analyses.

The specific research questions were: 1) What is the available evidence written in English, French, Chinese and/or Korean on the use of CAM in conjunction with male and female factor medical fertility treatment? 2) What evidence exists on using CAM for reducing psychological distress, including, stress, anxiety, and depression during men’s and women’s medical fertility treatment? 3) What is the quality of the evidence available on using CAM for improving fertility outcomes and fertility-related mental distress for both men and women experiencing infertility? Based on the review of the evidence, the conclusion contains recommendations about the potential benefits of particular CAM for both male and female fertility patients.

## Methods

### Type of review

A scoping review was conducted to compile and evaluate the evidence available for the use of CAM during fertility treatment. A scoping review is “a form of knowledge synthesis that addresses an exploratory research question aimed at mapping key concepts, types of evidence and gaps in research related to a defined area or field by systematically searching, selecting, and synthesizing existing knowledge” [13]. Scoping reviews help to provide information surrounding the effectiveness of treatments and are becoming increasingly important in providing information to determine evidence based-treatments (EBT) [14]. They are useful for reviewing topics where there is limited information and the methodologies used are disparate across studies [15]. The ability to comprehensively analyze information with various outcomes and measures offers an advantage over the more traditional systematic reviews such as meta-analyses. Scoping reviews also identify potential gaps in the evidence, pointing to areas where further research should be conducted [14, 15]. The stages set out by Arksey and O’Malley [14] were used to conduct a scoping review of the different types of CAM used in conjunction with infertility treatment: stage one identifies the research question; stage two identifies relevant studies; stage three involves study selection; stage four charts the data; stage five collates, summarizes and reports the results.

### Stage 1-identifying the research question

The research questions (see above) were identified while collecting evidence surrounding the effectiveness of CAM. During this process, it became clear that there was no comprehensive review of the effectiveness of CAM in relation to specific fertility outcomes.

### Stage 2-identifying relevant studies

On the basis of the National Center for Complementary and Alternative Medicine’s (NCCAM) review of popular CAM methods, the following methods were included: acupressure, acupuncture, Ayurveda, homeopathy, hypnosis, Chinese medicine, chiropractory, massage therapy, meditation, Mercier Therapy, mindfulness, naturopathy, relaxation, reflexology, reiki, touch therapy, yoga (see Table 1 for the search terms used and see Additional file 1 for definitions of methods). We categorized these common CAM methods according to the NCCAM classification system: alternative medicine systems (AMS; e.g. acupuncture), biological-based therapies (BBT; e.g. herbal supplements), manipulative and body-based therapies (MBBT; e.g. chiropractic care), and mind–body therapies (MBT; e.g. meditation) [8]. Although the NCCAM includes nutrition, diet and supplements in their definition of CAM, we chose not to include nutrition and supplements in our scoping review as dietary and nutritional advice is often viewed to be part of biomedical fertility treatments [16]. We did not include treatments such as Chelation therapy as its purpose is not intended for infertility. Some of the CAM methods identified by NCCAM like traditional healers were included by searching for “herbal medicine” rather than the title of the practitioner in an attempt to identify the mechanism of action (i.e., the herb). These common CAM methods were then combined with the search terms for our populations and outcomes of interest. Population terms included male infertility/subfertility/fertility, female infertility/subfertility/fertility. The outcomes of interest were emotional distress AND infertility/subfertility/fertility, anxiety AND infertility/subfertility/fertility, depression AND infertility/subfertility/fertility, infertility-related distress, IVF, ICSI, assisted reproduction, fertility treatment, and infertility treatment (see Table 1).

**Table 1:** Search Terms Used

Population Terms	Outcomes of Interest	Type of CAM
General infertility, subfertility, fertility	Type of Treatment assisted reproduction, fertility treatment, infertility treatment, IVF, ICSI	General CAM Terms alternative medicine, complementary alternative medicine, complementary medicine, alternative medicine
Female female infertility, female subfertility, female fertility	Mental Health anxiety, depression, infertility-related distress, emotional distress	Alternative Medicine Systems acupressure, acupuncture, moxibustion, naturopathy, homeopathy, Ayurveda, Traditional Chinese Medicine, traditional medicine
Male male infertility, male subfertility, male fertility		Biologically Based Therapies (BBT) Chinese herbal medicine, herbal medicine, herbal supplements
		Manipulative-and-Body Based Therapies (MBBT) chiropractory, healing touch, massage therapy, Mercier therapy, osteopathy, reflexology, reiki, shiatsu, therapeutic touch, touch therapy
		Mind-Body Therapies (MBT) hypnosis, meditation, relaxation, yoga

The CAM method, population terms and outcomes of interest were combined using “AND,” and searches were undertaken in the following databases: Cochrane Library, Medline Ovid, Pubmed, and PsycInfo.

#### Stage 3-study selection

**Fig. 1:**
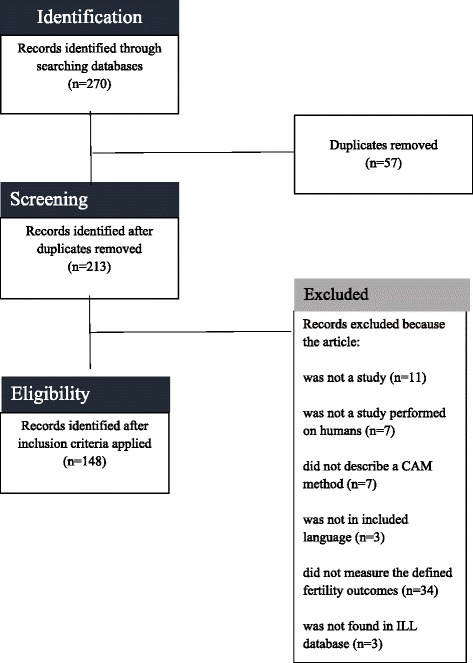
Exclusion Criteria

The search was limited to studies published from January 2006–June 2016. Both qualitative and quantitative articles were included for review. Articles were excluded if they were not written in English, Spanish, French, Chinese or Korean, if they did not contain data points that measured the specified outcomes (female fertility, male fertility and/or psychological distress), and/or if the full-text articles could not be located using the Colombo Interlibrary Loan system (see Fig. 1).

### Stage 4-charting the data

Each article was read independently by one of the authors. The following information was recorded about each article: language of the article and the abstract, type of CAM, type of study (see Table 2 for the types of studies included), the research question, the fertility outcome assessed, how the fertility outcome was assessed, the number of participants, the description of the control and treatment groups, the results including statistical significance of the findings (*p* < 0.05), the accuracy of the abstract (i.e. if the abstract contained correct findings and/or interpretation of results), and any bias (i.e. a prejudice in favor of the authors’ hypotheses and/or conflicts of interest reported or omitted by the study authors) (see Additional file 2). After this information was recorded, the first author assessed the level of evidence of each individual article using Australia’s National Health and Medical Research Council’s Evidence Hierarchy (NHMRC) [17, 18]. This tool was developed by a team of researchers and clinical practitioners to evaluate the clinical effectiveness of medical evidence (see Table 2). Level I evidence includes studies that are obtained from a systematic evaluation of randomized control trials; level II evidence is “evidence obtained from at least one properly-designed randomized trial;” level III-1 evidence is “evidence obtained from well-designed pseudorandomized controlled trials;” level III-2 evidence is “evidence obtained from comparative studies with concurrent controls and allocation is not randomized, cohort studies, case-control studies, or interrupted time series with a control group;” level III-3 is “evidence obtained from comparative studies with historical control, two or more single arm studies, or interrupted time series without a parallel control group;” and level IV evidence is “evidence obtained from case series, either post-test or pre-test/post-test” [18].

**Table 2:** Types of Studies by Evidence Level

Level of Evidence	Types of Studies
Level I	systematic review of level II studies, meta-analysis
Level II	randomized control trial
Level III-1	pseudorandomized control trial
Level III-2	comparative studies including non-randomized experimental trials, cohort studies, case-control studies, interrupted time series with controls
Level III-3	comparative studies without controls including historical control studies, two or more single-arm studies, interrupted time series without a parallel control group, cross-sectional
Level IV	case series, nonsystematic review, survey, qualitative interviews

## Results

A total of 148 out of 270 articles were determined to be relevant to review after applying the exclusion criteria (see Table 3). 101 articles were written in English, forty-four articles were written in Chinese with forty-three having an English abstract, and three articles were written in Korean all having English abstracts. Only one article of forty-four (2.3%) written in Chinese had an abstract that did not accurately describe the results while thirteen of the 101 (12.9%) articles in English had abstracts that did not accurately represent the findings of the study. All of the articles written in Korean had accurate abstracts. Articles that contained information about two methods (*n* = 17) were reviewed in both areas. The following results describe the rated evidence as per Australia’s National Health and Medical Research (NHRMC). The risk of bias was assessed for each article. Articles with lower levels of evidence had increased risk for author bias. Table 4 describes how many articles were found in relationship to each CAM area. No articles were found in relation to chiropractic medicine, reiki, reflexology, shiatsu, therapeutic touch therapy, or touch therapy.

**Table 3:** Number of Studies by CAM Method (*N* = 147)

Alternative Medicine Systems (AMS)	Biological Based Therapies (BBT)	Manipulative and Body-Based Therapies (MBBT)	Mind-Body Therapies (MBT)
Acupuncture (*n* = 44)^a^	Herbal Medicine (*n* = 16)^b^	Massage (*n* = 3)	Hypnosis (*n* = 2)
Ayurveda (*n* = 17)	Homeopathy (*n* = 1)	Osteopathy (*n* = 1)	Relaxation (*n* = 8)
Traditional Chinese Medicine (*n* = 71)^a,b^	Naturopathy (*n* = 1)	Chiropractic medicine, reiki, reflexology, shiatsu, therapeutic touch, touch therapy (*n* = 0)	Yoga (*n* = 3)

^a^There were 11 articles that contained evidence for both acupuncture and Traditional Chinese Medicine. Duplicate articles were reviewed in all categories

^b^There were 6 articles that contained evidence for both acupuncture and herbal medicine. Duplicate articles were reviewed in all categories

**Table 4:** Rating the Evidence

Method	Type of Outcome	Level I	Level II	Level III-1	Level III-2	Level III-3	Level IV
*Acupuncture*	*Female Fertility (n = 32)*	5 (15.6%)	12 (37.5%)	1 (3.1%)	3 (9.4%)	4 (12.5%)	7 (21.9%)
	*Male Fertility (n = 9)*	3 (33.3%)	1 (11.1%)	0 (0.0%)	0 (0.0%)	1 (11.1%)	4 (44.4%)
	*Mental Health (n = 8)*	1 (12.5%)	3 (37.5%)	0 (0.0%)	1 (12.5%)	1 (12.5%)	2 (25.0%)
*Ayurveda*	*Female fertility (n = 9)*	0 (0.0%)	3 (33.3%)	0 (0.0%)	0 (0.0%)	0 (0.0%)	6 (66.7%)
	*Male fertility (n = 8)*	0 (0.0%)	2 (25.0%)	1 (12.5%)	3 (37.5%)	0 (0.0%)	2 (25.0%)
	*Mental health (n = 1)*	0 (0.0%)	1 (100.0%)	0 (0.0%)	0 (0.0%)	0 (0.0%)	0 (0.0%)
*Chinese Herbal Medicine*	*Female fertility (n = 53)*	12 (22.6%)	25 (47.2%)	2 (3.8%)	3 (5.7%)	3 (5.7%)	8 (15.1%)
	*Male fertility (n = 17)*	2 (11.8%)	6 (35.3%)	7 (41.2%)	1 (5.9%)	0 (0.0%)	1 (5.9%)
	*Mental health (n = 2)*	0 (0.0%)	0 (0.0%)	0 (0.0%)	0 (0.0%)	0 (0.0%)	2 (100%)
*Herbal Medicine*	*Female fertility (n = 6)*	1 (16.7%)	0 (0.0%)	0 (0.0%)	0 (0.0%)	2 (33.3%)	3 (50.0%)
	*Male fertility (n = 10)*	2 (20%)	1 (10%)	0 (0.0%)	0 (0.0%)	1 (10%)	6 (60.0%)
	*Mental health (n = 1)*	0 (0.0%)	0 (0.0%)	0 (0.0%)	0 (0.0%)	1 (100%)	0 (0.0%)
*Homeopathy*	*Female fertility (n = 1)*	0 (0.0%)	0 (0%)	0 (0.0%)	0 (0.0%)	0 (0.0%)	1 (100%)
*Hypnosis*	*Female fertility (n = 2)*	0 (0.0%)	0 (0.0%)	0 (0.0%)	1 (50.0%)	0 (0.0%)	1 (50.0%)
*Massage*	*Female fertility (n = 3)*	0 (0.0%)	1 (33.3%)	1 (33.3%)	0 (0.0%)	0 (0.0%)	1 (33.3%)
*Naturopathy*	*Female fertility (n = 1)*	0 (0.0%)	0 (0.0%)	0 (0.0%)	0 (0.0%)	0 (0.0%)	1 (100%)
*Osteopathy*	*Female fertility (n = 1)*	0 (0.0%)	0 (0.0%)	0 (0.0%)	0 (0.0%)	0 (0.0%)	1 (100%)
*Relaxation*	*Mental health (n = 8)*	0 (0.0%)	1 (12.5%)	1 (12.5%)	3 (37.5%)	0 (0.0%)	3 (37.5%)
*Yoga*	*Mental health (n = 3)*	0 (0.0%)	0 (0.0%)	0 (0.0%)	1 (33.3%)	0 (0.0%)	2 (66.7%)

*n* = number of studies, CAM methods which had no studies (*n* = 0) evaluating specific fertility outcomes were removed from the table; n.b. Articles could evaluate multiple outcomes (e.g. some articles that evaluated female fertility outcomes also evaluated male fertility outcomes)

### Acupuncture

Forty-four articles were reviewed with evidence ranging from level I to level IV. Some articles evaluated multiple outcomes. Five articles were written in Chinese, one article was written in Korean, and thirty-eight were written in English. Eight of the forty-four studies (18.2%) were rated as having level I evidence (with one study assessing multiple outcomes). Five level I studies showed an improvement in fertility outcomes: two showed an improvement in female fertility, two showed an improvement in male fertility, and two showed an improvement in mental health (see Table 4). The number of studies of acupuncture that had level II evidence was fifteen of forty-four (34.1%). Seven of the twelve (58%) level II studies evaluating acupuncture in relationship to female fertility showed an improvement in outcome while the only level II study evaluating male fertility showed an improvement in outcome (see Table 5). Additionally, the three level II studies evaluating mental health outcomes with the use of acupuncture showed an improvement in outcomes (see Table 5). The remaining twenty-two studies ranged in their levels of evidence from level III-1 to level IV with a minority showing improvement in outcomes (see Table 4).

**Table 5:** Evidence for Positive Outcomes

Method	Type of Outcome	Level I Improved Outcome	Level II Improved Outcome	Level III-1 Improved Outcome	Level III-2 Improved Outcome	Level III-3 Improved Outcome	Level IV Improved Outcome
*Acupuncture*	*Female Fertility*	40% (2/5)	58% (7/12)	0% (0/1)	33.3% (1/3)	100% (4/4)	(14.3%) 1/7
	*Male Fertility*	66.7% (2/3)	100% (1/1)	0% (0/1)	-- (0/0)	100% (1/1)	100% (4/4)
	*Mental Health*	100% (1/1)	100% (3/3)	-- (0/0)	0% (0/1)	0% 0/1	100% (2/2)
*Ayurveda*	*Female Fertility*	-- (0/0)	67% (2/3)	-- (0/0)	-- (0/0)	-- (0/0)	100% (6/6)
	*Male Fertility*	-- (0/0)	100% (2/2)	100% (1/1)	66.7% (2/3)	-- (0/0)	100% (2/2)
	*Mental Health*	-- (0/0)	100% (1/1)	-- (0/0)	-- (0/0)	-- (0/0)	-- (0/0)
*Chinese Herbal Medicine*	*Female fertility*	75% (9/12)	84% (21/25)	100% (2/2)	66.7% (2/3)	66.7% (2/3)	37.5% (3/8)
	*Male fertility*	100% 2/2	100% (6/6)	85.7% (6/7)	100% (1/1)	-- (0/0)	100% (1/1)
	*Mental health*	-- (0/0)	-- (0/0)	-- (0/0)	-- (0/0)	-- (0/0)	100% (2/2)
*Herbal Medicine*	*Female fertility*	0% (0/1)	-- (0/0)	-- (0/0)	-- (0/0)	100% (2/2)	66.7% (2/3)
	*Male fertility*	50% (1/2)	100% (1/1)	-- (0/0)	-- (0/0)	100% (1/1)	66.7% (4/6)
	*Mental health*	-- (0/0)	-- (0/0)	-- (0/0)	-- (0/0)	0% (0/1)	-- (0/0)
*Homeopathy*	*Female fertility*	-- (0/0)	-- (0/0)	-- (0/0)	-- (0/0)	-- (0/0)	100% (1/1)
*Hypnosis*	*Female fertility*	-- (0/0)	-- (0/0)	-- (0/0)	100% (1/1)	-- (0/0)	100% (1/1)
*Massage*	*Female* *fertility*	-- (0/0)	100% (1/1)	100% (1/1)	-- (0/0)	-- (0/0)	100% (1/1)
*Naturopathy*	*Female* *fertility*	-- (0/0)	-- (0/0)	-- (0/0)	-- (0/0)	-- (0/0)	100% (1/1)
*Osteopathy*	*Female fertility*	-- (0/0)	-- (0/0)	-- (0/0)	-- (0/0)	-- (0/0)	100% (1/1)
*Relaxation*	*Mental health*	-- (0/0)	100% (1/1)	100% (1/1)	100% (3/3)	-- (0/0)	100% (3/3)
*Yoga*	*Mental health*	-- (0/0)	-- (0/0)	-- (0/0)	100% (1/1)	-- (0/0)	100% (2/2)

Fractions in parenthesis represent the number of studies that improve the outcome over the total number of studies in that area. CAM methods which had no studies (*n* = 0) evaluating specific fertility outcomes were removed from the table

### Ayurveda

Eighteen articles were reviewed with evidence ranging from level II to level IV. Some articles examined multiple outcomes. All articles were written in English. Six of the eighteen studies (33.3%) were rated as having level II evidence. All five (100%) of the level II studies showed an improvement in fertility outcomes: three showed an improvement in female fertility, two showed an improvement in male fertility, and one showed an improvement in mental health (with one study evaluating two outcomes) (see Table 5). There was only one study that had level III-1 evidence which showed an improvement in male fertility outcomes. While there were three level III-2 studies, only two showed an improvement in male fertility outcomes (see Table 5). The remaining eight studies were level IV studies with six studies showing an improvement in female fertility and two studies showing an improvement in male fertility (see Table 4). The risk of bias for female fertility outcomes was very high as study protocols were not standardized.

### Chinese herbal medicine

Seventy-two articles were reviewed with evidence ranging from level I to level IV. Some articles examined multiple outcomes. Forty-two articles were written in Chinese with thirty-six of those articles providing an English abstract. Twenty-nine articles were written in English. Fourteen of the seventy-two articles (19.4%) were level I studies with twelve (16.6%) evaluating female fertility outcomes and two (2.7%) rating male fertility outcomes. Nine of these level I studies showed an improvement in female fertility outcomes and two showed an improvement in male fertility outcomes (see Table 5). Thirty-one of the seventy-two articles (43.1%) were rated as having level II evidence; twenty-one of the thirty-one articles (67.8%) showed an improvement in female fertility outcomes and six showed an improvement in male fertility outcomes (see Table 5). There were only two articles that evaluated mental health outcomes. Although both studies showed an improvement in mental health outcomes, the evidence for this improvement was very low (level-IV).

### Herbal medicine

Seventeen articles were reviewed with evidence ranging from level I to level IV. Some articles examined multiple outcomes. All articles were written in English. Three of the seventeen articles (17.6%) were level I studies with one evaluating female fertility outcomes and two evaluating male fertility health outcomes (see Table 5). The majority of the studies (nine out of seventeen articles, 52.9%) evaluating herbal medicine had the lowest level of evidence. Two of the three (66.7%) level IV studies that evaluated female fertility outcomes showed an improvement in outcome while four of the six (66.7%) level IV studies that evaluated male fertility outcomes showed an improvement in outcome. The only study related to mental health was rated as having level III-3 evidence and it showed an improvement in outcomes.

### Homeopathy

There was only one article found that evaluated the use of homeopathy. It was written in English, and examined female fertility outcomes. While the study showed an improvement in outcomes, it was rated as having the lowest level of evidence, level IV (see Tables 4 and 5). There was a high risk of bias in this study as there was not standardized recruitment or predefined outcomes.

### Hypnosis

There were two articles found that evaluated the use of hypnosis and fertility treatment. They were both written in English and examined female fertility outcomes. One study was rated as having level III-2 evidence and the other as having level IV evidence (see Table 4). Both showed an improvement in outcomes (see Table 5). The risk of bias is moderate as the study did have a control group, but it is uncertain whether or not the effects could be replicated.

### Massage

There were three articles found that evaluated the use of massage and fertility outcomes. They were all written in English and evaluated female fertility outcomes. One study had level II evidence, one had level III-1 evidence and one had level IV evidence (see Table 4). All showed an improvement in outcomes. All studies had a high risk of bias as there was a lack of a real control group and nonstandardized definitions of fertility were used.

### Naturopathy

There was only one article found that evaluated the use of naturopathy through a nonsystematic review. It was written in English, and examined female fertility outcomes. While the review showed an improvement in outcomes, it was rated as having the lowest level of evidence, level IV (see Tables 4 and 5). This nonsystematic review has a high risk for bias as it was unclear how the articles were found.

### Osteopathy

There was only one article found that evaluated the use of osteopathy. It was written in English, and examined female fertility outcomes. While the study showed an improvement in outcomes, it was rated as having the lowest level of evidence, level IV (see Tables 4 and 5). Since the observational case study was written, performed and qualitatively evaluated by the sole author of the publication, the results have a high risk for bias.

### Relaxation

There were eight studies that evaluated the use of relaxation techniques and fertility outcomes. All articles were written in English, and examined mental health outcomes. The studies ranged in levels of evidence from level II to level IV with level III-2 and level IV having three articles each (see Table 4). All studies showed an improvement in mental health outcomes.

### Yoga

There were three studies that evaluated the use of yoga and fertility outcomes. All articles were written in English, and examined mental health outcomes. All three articles showed an improvement in mental health outcomes with one article having level III-2 evidence while the two articles had level IV evidence.

## Discussion

While numerous studies (*n* = 148) examined the use of CAM in relation to female fertility, male fertility and/or mental health outcomes, the body of evidence is not evenly distributed amongst different types of CAM methods. Most studies focused on acupuncture and Chinese Herbal Medicine. Both acupuncture and Chinese Herbal Medicine are considered Alternative Medicine Systems (AMS) by the National Center for Complementary and Alternative Medicine (NCCAM). However, there was much less evidence for biological-based therapies (BBTs), manipulative and body based therapies (MBBTs), and mind-body therapies (MBT) (see Table 3). Additionally, female fertility outcomes were evaluated more frequently than both male fertility outcomes and mental health outcomes with the least amount of evidence for alternative treatments (e.g. treatments outside of psychotherapy or cognitive based therapy) in relation to mental health outcomes.

There was a large body of evidence found written in Chinese for both acupuncture and Chinese Herbal Medicine (*n* = 44). There was a smaller body of evidence written in Korean (*n* = 3). Most of these articles had English abstracts (*n* = 47); in all but one case, the English abstract contained information that was consistent with the results presented in the body of the paper. This finding suggests that researchers could safely rely on abstracts written in English to capture the findings of studies written in languages that they do not read. Attention should be given to English abstracts where the article is also written in English as there was a certain number of English abstracts for English-language papers (13/101, 12.9%) which contained inaccurate information such as incorrect reporting of sample size or results that suggested a more positive outcome than what was presented in the discussion. If possible, researchers and clinicians assessing the evidence provided by a particular study should verify that the abstract and full-text are congruent.

Studies that discussed acupuncture and fertility treatment were the most numerous and had the highest levels of evidence, especially for female and male fertility outcomes. While there was some level I evidence that showed that acupuncture did improve female and male fertility outcomes, there is still no conclusive evidence that the use of acupuncture in conjunction with conventional fertility treatment will improve fertility outcomes because not all studies showed improvements in outcomes and for some outcomes (e.g. mental health) there were few studies with high levels of evidence (see Table 6). Many treatments had lower levels of evidence including homeopathy, hypnosis, massage, naturopathy, osteopathy, relaxation and yoga. In addition to the low levels of evidence, there were few studies surrounding these CAM treatments. While there is a smaller body of evidence surrounding Ayurvedic medicine and male factor fertility outcomes, the existing evidence is promising for outcomes such as improving sperm parameters in men. Additionally, the large body of mostly positive evidence for the use of Chinese Herbal Medicine (CHM) for female factor infertility may suggest that Chinese herbs could be used as an effective supplement; however, more meta-analyses of the current randomized control trials are needed in order to gain a more adequate understanding of the particular type of female factor infertility CHM could promote.

**Table 6:** Overall Summary of CAM Outcomes

Type of CAM	Female Factor	Male Factor	May reduce stress
Acupuncture	?	?	?
Ayurveda	X	?	X
Chinese Herbal Medicine	?	X	X
Herbal Medicine	X	X	X
Chiropractic Medicine	X	X	X
Massage	X	X	✓
Osteopathy	X	X	X
Homeopathy	X	X	X
Naturopathy	X	X	X
Hypnosis	X	X	X
Yoga	X	X	✓
**Key**	**Level of Evidence**		
X	There is a lack of good scientific evidence that this CAM technique improves the outcome.		
?	Evidence is conflicting. While some studies show that there is an improved outcome, others show no change in outcome.		
✓	There is evidence that this CAM technique may improve the outcome.		

There were few studies that assessed mental health outcomes. While many of these studies do show an improvement in mental health outcomes, the studies that do exist are of low quality and have high risks for bias. This lack of assessment is problematic because CAM practitioners promote the use of CAM as a holistic treatment to improve “emotional outcomes” and promote “healthy lifestyles” [19]. The evidence that exists generally focuses on improved measures of perceived stress and depression, rather than employing standardized scales assessing quality of life during fertility treatment (i.e FertiQoL). Future research should consider quality of life as an outcome of CAM in order to capture how the use of CAM may improve a person’s overall well-being during fertility treatment.

Overall, there was a lack of level I and level II studies in evaluating specific CAM therapies in relation to fertility treatment. This may be due to lack of funding provided to CAM researchers, and also due to the difficulty conducting randomized control trials for treatments that require holistic and individualistic approaches. For example, in acupuncture there is a debate about which type of placebo to use (i.e. sham at acupoints, sham at nonacupoints, no intervention, etc.), and doses are often individualized [20]. These individual differences in study design and treatment protocol have led to the development of standardized ways of evaluating and performing control-trials for acupuncture (i.e. *STRICTA*), [21], Chinese herbal medicine [22], and herbal medicine, [23]; however, guidelines for reporting other CAM methods are less standardized. This results in a variety of ways of performing and reporting trial outcomes, which creates difficulty in comparing data across studies. Additionally, most articles (outside of RCTs) suggested a possible placebo effect where individuals who believed the CAM treatments were helping were more likely to have improved outcomes.

The mechanisms of actions of the variety of CAM techniques are largely unknown. It was difficult to determine how each specific CAM technique affected the specific outcome measured as within various CAM methods there are different types of interventions used. For example, in Ayurvedic medicine, oils were used to attempt to clear fallopian tubes while in other cases roots were given in order to improve pregnancy rates. Additionally, the studies analyzed did not necessarily investigate the mechanism that may have caused improvement, but were more interested in the outcome itself. Future studies of CAM should consider the mechanism of action of these methods to produce better quality of evidence.

### Strengths and limitations

A scoping review of the literature was performed to evaluate twelve different CAM methods. The review was not confined to the English literature but rather included articles that were written in four different languages. Although the individual study results were not statistically combined, the collating of various outcomes allows for a determination of which methods may improve fertility outcomes and/or need more statistical evidence. The article also evaluates multiple CAM methods, some of which were not previously systematically reviewed (e.g. hypnosis).

This scoping review was only performed on published studies. Therefore, we may have a publication bias towards positive results, suggesting that the results of our scoping review be interpreted with caution. There may also have been articles that our English search terms missed that were not evaluated. Additionally, we were not able to have multiple authors read the individual articles to determine the accuracy of the levels of evidence as we only had one author who could read and interpret the Chinese articles. However, author one did confirm that the type of study written by each author correctly matched the level of evidence. When the type of study was unclear, authors conferred about the type of study and the level of evidence was interpreted conservatively. Since there was a broad variety of fertility outcomes identified, sometimes with conflicting definitions, the review could not both assess the entire body of evidence and the individual fertility outcomes identified by every article.

### Future research directions

This scoping review has highlighted the lack of strong evidence for CAM methods to improve fertility outcomes. However, this lack of evidence should not mean that no future studies be performed on CAM and fertility outcomes. We suggest that future research should look towards the most promising areas of CAM (i.e. Ayurveda for male factor infertility, Chinese Herbal Medicine for female fertility and relaxation techniques for promoting mental health during fertility treatment), and subsequently, implement studies with more purposeful and valid research designs in order to form conclusions that are based on higher levels of evidence. In order to discourage publication bias, publishers should also be willing to publish evidence that shows negative or no effects as this is important evidence in determining the effectiveness of CAM. In other words, scientists and practitioners of CAM should more systematically research CAM therapies that have already been proven to be effective (e.g. relaxation techniques for improving mental health outcomes). Additionally, more standard definitions should be applied to articles assessing fertility outcomes so that results are statistically comparable across studies. For example, we suggest that measurements of improvement in female fertility outcomes should follow the BESST (Birth Emphasizing a Successful Singleton at Term) endpoints, which define assisted reproductive success based on live full-term singleton births [24].

## Conclusions

There is a lack of evidence for the use of CAM to improve fertility outcomes as most techniques are not evaluated by studies that produce high levels of evidence (i.e. level I and level II). Additionally, across fertility outcomes and CAM techniques, studies do not always show a significant improvement in fertility outcomes. Rather, the overall evidence points to CAM having no significant effect on the fertility outcome of interest. Although there has been an increase in the use of CAM use over time [25], despite the fact that this increase is not necessarily supported by empirical evidence. However, some individuals may choose to use CAM methods to feel more in control of their fertility treatment [5]. This potential feeling of empowerment, however, is not adequately captured by the existing studies involving CAM and mental health as most of the mental health studies found were of poor quality, and did not measure the quality of life of the participants or their feelings of empowerment. Thus, there remain questions as to whether CAM does improve fertility patients’ quality of life and/or their feelings of control over treatment.

Fertility physicians should inform their patients about the lack of standardized empirical evidence for all CAM methods with the realization that patients may choose to engage in CAM despite the lack of evidence. Since no methods were found to negatively impact fertility or mental health outcomes, there should be less concern with patients engaging in CAM that may hinder their fertility outcomes. However, physicians and patients should consider the evidence base for treatments that claim to significantly improve fertility and/or mental health in order to manage expectations regarding their efficacy. It should also be acknowledged that patients may choose to undergo a particular CAM procedure because it helps them feel more empowered in their treatment. The widespread use of CAM among fertility patients should provide an impetus for the design and implementation of high-quality studies of their effects.

## Additional files

Additional file 1: Definitions of CAM Methods-Definition of CAM methods analyzed. (DOCX 14 kb)

Additional file 2: CAM Review Sheet-Copy of the Google review sheet used to record the information from the articles. (PDF 90 kb)

## Abbreviation

AMS: Alternative medicine systems
BBT: Biological-based therapies
CAM: Complementary and alternative medicine
CDC: Center for Disease Control
CHM: Chinese Herbal Medicine
IVF: In vitro fertilization
MBBT: Manipulative and body-based therapies MBT Mind-body therapies
NCCAM: National Center for Complementary and Alternative Medicine
NHMRC: National Health and Medical Research Council

## Footnotes

1 Rayner, Willis and Burgess [4] do not provide a definition of how religious intervention and spiritual healing differ. We have listed them as separate types of CAM to reflect the findings that Rayner, Willis and Burgess [4] present.
