# Supplementary material for: Evidence for the use of complementary and alternative medicines during fertility treatment: a scoping review
Source: BMC Complement Altern Med. 2018 May 15;18:158. doi: 10.1186/s12906-018-2224-7 (PMC5952848; doi:10.1186/s12906-018-2224-7)
Supplement: Supplementary file 1 — Definitions of CAM Methods-Definition of CAM methods analyzed. (DOCX 14 kb) [file 12906_2018_2224_MOESM1_ESM.docx]

**Supplementary Material A: Definitions of CAM Methods^[[1]](#footnote-1)^**

Acupuncture—A procedure that most often involves the use of small needles for penetration that are manipulated by the hands of the practitioners or electronically. It is one of the key components of traditional Chinese Medicine, but because of its popularity it was assessed as a separate method.

Ayurveda—A form of medicine that originated in India. Herbs, massage and special diets are often used as treatment.

Chinese Herbal Medicine—A form of medicine that originated in China. Herbs and acupuncture are commonly used as treatment. In this paper acupuncture was used to treat a variety of conditions.

Herbal medicine—Medicine that relies on herbs and botanicals for treatment. For the purpose of this study, Chinese herbal medicine was assessed separately.

Chiropractic medicine—Focuses on the relationship between the body’s structure and health. Generally, involves the manipulation of the spine and other parts of the body to adjust for alignment.

Massage—Therapy that generally involves manipulation of the body’s tissue through pressing and rubbing.

Osteopathy—A form of medicine that relies on manipulation of the muscles, joints and bones for treatment.

Homeopathy—An alternative form of treatment that relies on the dilution of substances. It is based on the principle “like cures like.”

Naturopathy—System of medicine that relies on a variety of dietary and lifestyle changes. It sometimes incorporates herbs, manipulative therapies, and detoxification strategies.

Hypnosis—Also called hypnotherapy. It is a mind-body technique that alters perception.

Yoga—A mind body practice that is rooted in Indian philosophy. It generally involves physical postures, a focus on the breath and a meditation component.

1. All definitions are based on the National Center of Complementary and Alternative Medicine’s definitions for CAM methods. [↑](#footnote-ref-1)
