# Supplementary material for: Evidence for the use of complementary and alternative medicines during fertility treatment: a scoping review
Source: BMC Complement Altern Med. 2018 May 15;18:158. doi: 10.1186/s12906-018-2224-7 (PMC5952848; doi:10.1186/s12906-018-2224-7)
Supplement: Supplementary file 2 — CAM Review Sheet-Copy of the Google review sheet used to record the information from the articles. (PDF 90 kb) [file 12906_2018_2224_MOESM2_ESM.pdf]

CAM review sheet

To analyze the validity and evidence of CAM methods

* Required

1. **Full APA citation** *

____

2. **Language of article** *

Mark only one oval.

- ( ) English
- ( ) French
- ( ) Chinese
- ( ) Chinese with English Abstract
- ( ) Korean

3. **Type of CAM** *

Check all that apply.

- [ ] Acupuncture
- [ ] Ayurveda
- [ ] Chinese Medicine (Traditional or Herbal)
- [ ] Chiropractory
- [ ] Herbal Medicine (Not Chinese)
- [ ] Homeopathy
- [ ] Hypnotheraphy/hypnosis
- [ ] Kinesiology
- [ ] Massage
- [ ] Meditation
- [ ] Naturopathy
- [ ] Osteopathy
- [ ] Relaxation
- [ ] Reflexology
- [ ] Yoga

4. **If drugs/preparations were used, which ones?**

5. **Type of Study** *
Mark only one oval.

- Meta-analysis
- Systematic Review of randomized control trials
- Nonsystematic Review
- Randomized Controlled Trial
- Pseudorandomized Controlled Trial
- Non-Randomized Experimental Trials
- Case-Controlled Study
- Retrospective and Prospective cohort Study
- Interrupted Time-Series with Controls
- Interrupted Time Series without Controls
- Historical control studies
- Two or more single-arm studies
- Case Series
- Survey
- Qualitative Interviews
- Opinion
- Other:

Characteristics of Sample

6. **N= (population size)**

7. **Description of control group (if any); n, what type of control**

8. **Description of treatment group (n=)** *

Research Question

9. **What is the research question?** *

10. **What is the outcome measured?** *
Check all that apply.

- [ ] Female Factor Infertility
- [ ] Male Factor Infertility
- [ ] Mental Health
- [ ] Other: ______

11. **How did they measure the outcome?** *

Check all that apply.

- [ ] Sperm count
- [ ] Sperm morphology
- [ ] Sperm concentration/volume
- [ ] Sperm Motility
- [ ] Live birth rate
- [ ] Ovulation
- [ ] Implantation Rates
- [ ] Pregnancy rate
- [ ] Miscarriage Rate
- [ ] Number of oocytes retrieved
- [ ] Spontaneous Abortion Rate
- [ ] Depression levels
- [ ] Anxiety levels
- [ ] Stress levels
- [ ] Fertility-related (di)stress
- [ ] Quality of life
- [ ] Marital satisfaction
- [ ] Other: ____________________

Findings

12. **Did the study find that the treatment improved the fertility outcome(s)?** *

Mark only one oval.

- ( ) Yes, improved the outcome
- ( ) No, it did not improve the outcome (i.e. no change)
- ( ) No, it had a negative impact on the outocme (e.g. resulted in a worsening of the outcome)
- ( ) Mixed results (please explain below)
- ( ) Unclear, the article does not have a clear conclusion (please explain below)

13. **If mixed results or unclear above, please explain here.**

____
____
____
____
____

14. **How much did the study affect the outcome? (give percentages, changes in odds-ratios with p-values if available)** *

15. **Was statistical significance achieved? (p value less than or equal to .05)** *
Mark only one oval.

- Yes
- No
- Marginal significance achieved (p value less than or equal to .10
- The study does not report p values

Abstract

16. **Does the article support the abstract?** *
Mark only one oval.

- Yes
- No

17. **If no, what are the differences between the article and the abstract?**

Conflict of Interest

18. **Do the author(s) report any conflict of interests?** *

19. **Do you see any conflicts of interests?**

Additional Information

20. **Do you think that the study is valid?** *

Mark only one oval.

- Yes
- No

21. **What problems, if any, do you see in this article?**

Powered by
Google Forms
